# Reaction of [^18^F]Fluoride at Heteroatoms and Metals for Imaging of Peptides and Proteins by Positron Emission Tomography

**DOI:** 10.3389/fchem.2021.687678

**Published:** 2021-06-23

**Authors:** Kymberley R. Scroggie, Michael V. Perkins, Justin M. Chalker

**Affiliations:** Institute for Nanoscale Science and Technology, College of Science and Engineering, Flinders University, Adelaide, SA, Australia

**Keywords:** fluorine-18, positron emission tomography, protein modification, aqueous fluorination, radiolabeling (18F)

## Abstract

The ability to radiolabel proteins with [^18^F]fluoride enables the use of positron emission tomography (PET) for the early detection, staging and diagnosis of disease. The direct fluorination of native proteins through C-F bond formation is, however, a difficult task. The aqueous environments required by proteins severely hampers fluorination yields while the dry, organic solvents that promote nucleophilic fluorination can denature proteins. To circumvent these issues, indirect fluorination methods making use of prosthetic groups that are first fluorinated and then conjugated to a protein have become commonplace. But, when it comes to the radiofluorination of proteins, these indirect methods are not always suited to the short half-life of the fluorine-18 radionuclide (110 min). This review explores radiofluorination through bond formation with fluoride at boron, metal complexes, silicon, phosphorus and sulfur. The potential for these techniques to be used for the direct, aqueous radiolabeling of proteins with [^18^F]fluoride is discussed.

## Introduction

Positron emission tomography (PET) is a powerful, minimally invasive molecular imaging technique. First used to detect brain tumors prior to surgical removal in the 1950s (Sweet, 1951; Wrenn et al., 1951), it is now used in the study of cardiac diseases (Robinson and Bourque, 2019) and myocardial perfusions (Driessen et al., 2017), neurodegenerative diseases such as Parkinson’s (Walker et al., 2018), Alzheimer’s (Chandra et al., 2019) and Huntington’s (Cybulska et al., 2020) diseases, inflammatory diseases (Wu et al., 2013), cancers including those that affect the breasts (Dijkers et al., 2009; Kurihara et al., 2015), lungs (De Ruysscher et al., 2012), skin (Duncan et al., 2016) and prostate (Jadvar, 2013), as well as diseases caused by bacterial infections (Auletta et al., 2019).

PET is primarily based on radiolabeled small-molecule probes that serve as imaging agents. These imaging agents, radiolabeled with a radionuclide that decays *via* β^+^ decay, release a positron (β^+^) which upon collision with an electron in the surrounding matter results in annihilation. In this process, the positron and electron are converted into a pair of photons with energies of 511 keV which are emitted at *ca.* 180° apart. Coincidence detection of these photon pairs allows the point at which annihilation occurred to be determined (Levin, 2005) giving an approximation of the temporal and spatial distribution of the imaging agent (Ametamey et al., 2008) and functional information about the biological processes occurring within the body.

There is a suite of radionuclides that decay *via* β^+^ decay with some more suited to PET studies than others (Blower, 2015). In general, those that have a low maximum positron energy are preferred as a radionuclide’s positron energy is directly related to the positron’s range—the distance it travels from the nucleus before it undergoes annihilation. Thus, for radionuclides with higher positron energies, there is a higher probability that annihilation will occur further away from the nucleus resulting in a lower spatial resolution (Levin and Hoffman, 1999). Additionally, the half-life of the radionuclide must also be adequate for the application and it is often matched to the biological half-life of the molecular probe. For example, larger biomolecules have slower pharmacokinetics and radionuclides with longer half-lives such as [^68^Ga]gallium (67.7 min), [^18^F]fluorine (110 min), [^64^Cu]copper (12.7 h) and [^124^I]iodine (4.18 days) are more suitable when they are to be used as imaging agents (Tolmachev and Stone-Elander, 2010; Warnders et al., 2018). The properties of some radionuclides that decay *via* β^+^ decay are illustrated in Table 1 (Brookhaven National Laboratory; Laboratoire National Henri Becquerel; Le Loirec and Champion, 2007a; Le Loirec and Champion, 2007b; Le Loirec and Champion, 2007c).

**TABLE 1:** Properties of positron emitting radionuclides.^a^

Radionuclide	β^+^ decay	Max β^+^ energy (MeV)	Mean β^+^ range (mm in water)	Half-life	Production
Carbon-13	99.8%	0.960	1.27^b^	20.4 min	^14^N(p,α)^11^C
Nitrogen-13	99.8%	1.20	1.73^b^	9.97 min	^16^O(p,α)^13^N
Oxygen-15	99.9%	1.73	2.97^b^	2.03 min	^14^N(d,n)^15^O
Fluorine-18	96.8%	0.634	0.66^b^	110 min	^18^O(p,n)^18^F
Scandium-44	94.3%	1.47	2.46^b^	3.97 h	^44^Ti/^44^Sc generator
Copper-64	17.5%	0.653	0.56^c^	12.7 h	^64^Ni(n,p)^64^Cu
Gallium-68^e^	88.9%	1.90	3.56^b^	67.7 min	^68^Ge/^68^Ga generator
Rubidium-82^e^	95.4%	3.38	7.49^b^	1.26 min	^82^Sr/^82^Rb generator
Zirconium-89	22.8%	0.902	1.27^c^	78.4 h	^89^Y(p,n)^89^Zr
Iodine-124^e^	22.7%	2.14	—	4.18 days	^124^Te(p,n)^124^I

a Laboratoire National Henri Becquerel, http://www.nucleide.org/Laraweb/index.php; Brookhaven National Library, https://www.nndc.bnl.gov/nudat2/.

b Le Loirec and Champion, 2007a.

c Le Loirec and Champion, 2007b.

d Le Loirec and Champion, 2007c.

e non-pure positron emitter; maximum positron energy (MeV) representative of most frequently emitted positron.

Central to the advancement of molecular imaging with PET is the development of suitable PET imaging agents. With growing opportunities to use PET for molecular diagnostic and therapeutic procedures (Ametamey et al., 2008) this has become a key area of research at the interface of medicine, chemistry and biology. Finding suitable molecular probes that can be readily labeled with positron emitting radionuclides, however, still represents one of the most significant challenges faced by the research community (Niwa and Hosoya, 2020).

This review is designed to be a historical perspective of the development of fluorine-18 probes for PET through heteroatom- and metal-fluorine bond formation, with specific emphasis on applications for PET imaging of peptides and proteins. [^18^F]fluoride labeling at boron, selected metals, silicon, phosphorous, and sulfur is discussed. And while there has been encouraging progress on late-stage C-F bond formation on peptides (Becaud et al., 2009; Roehn et al., 2009; Jacobson et al., 2011), these reactions typically require elevated temperatures and organic solvents not compatible with proteins. Therefore, C-F bond formation will not be discussed in detail in this review. Rather, key foundational studies on forming bonds between fluorine and heteroatoms and metals are examined as well as recent progress in using these techniques to label peptides and proteins, especially at a late stage for PET imaging. Discussion of using these transformations in aqueous media and under protein compatible conditions are highlighted where possible, to emphasize the challenges for direct labeling of proteins with [^18^F]fluoride.

## Background

### Biomarkers as Diagnostic Imaging Agents in Positron Emission Tomography

Biomarkers have been essential to our current understanding of disease and to our endeavor to improve human health. Described as “a characteristic that is objectively measured and evaluated as an indicator of normal biological processes, pathogenic processes, or pharmacological responses to a therapeutic intervention” (Biomarkers Definitions Working Group, 2001), biomarker is a relatively new formal term. The use of biomarkers in medicine, however, has long been part of clinical care. Blood glucose levels for example have long been used as a biomarker for diabetes (Pirart, 1978) as has cholesterol levels to assess cardiovascular risk (Ignatowski, 1909) and the presence of antibodies as an indicator of infection (Waldmann, 1991).

Biomarkers that indicate pathogenic processes are particularly useful as PET imaging agents for the detection, characterization and staging of disease. The most prominent imaging agent in PET is 2-[^18^F]fluoro-deoxy-d-glucose ([^18^F]FDG), which is used to study glucose metabolism (Reivich et al., 1979). [^18^F]FDG has found extensive use in oncology as tumor tissues metabolize glucose at a higher rate than healthy tissues, and in neurology where diminished glucose uptake can signify the onset of neurological diseases (Gambhir et al., 2001). Another is 6-[^18^F]fluoro-3,4-dihydroxyphenylalanine ([^18^F]DOPA) used to study the nigrostriatal dopaminergic pathway (Garnett et al., 1983). [^18^F]DOPA is the standard probe for staging in Parkinson’s disease (Gharibkandi and Hosseinimehr., 2019).

Small molecules have always held a privileged status as PET probes, primarily because they are simpler to radiolabel than macromolecules. However, interest in using larger probes such as peptides and proteins has grown substantially of late (Richter and Wuest, 2014). The most attractive properties of peptide and protein imaging agents are that they often exhibit high binding affinities and specificities for their biomarker targets (Morris et al., 2019). With recent advances in molecular display techniques, we now have increasingly large amounts of information available regarding peptide-protein and protein-protein interactions that are associated with pathogenic process. Furthermore, these advances now allow us to select and evolve peptides and proteins which have higher affinities for their molecular targets than their natural analogues (Tolmachev and Stone-Elander, 2010). By translating this knowledge to the development of new imaging agents and pairing it with the sensitivity of PET, peptides and proteins have the potential to be highly useful imaging agents in the early detection, staging and unambiguous diagnosis of disease.

### Fluorine-18

Of the positron emitting radionuclides, fluorine-18 is most often used for PET. Possessing favorable nuclear properties, it has a moderate half-life of 110 min, a low maximum positron energy at 0.634 MeV and it is a pure positron emitter decaying *via* β^+^ decay 96.7% of the time and electron capture the remaining 3.1%. Thanks in large part to the wide use of [^18^F]FDG, [^18^F]fluoride is now readily available with the production facilities and logistics in place for its transport to centers without a cyclotron (Tolmachev and Stone-Elander, 2010).

Fluorine-18 also possesses favorable chemical and electronic properties. Similar to replacement of non-radioactive carbon with the positron emitting isotope carbon-11, non-radioactive fluorine can be replaced with fluorine-18 with negligible effects on the biological properties and activity of the molecule. While there are a limited number of fluorine atoms found in biological molecules, there are a number of hydrogens and hydroxyl groups. Similar in its size to hydrogen (van der Waals radii fluorine 1.47 Å, hydrogen 1.20 Å) and in electronic nature to the hydroxyl group, fluorine can serve as a bioisostere for either group. A classic example of the former is [^18^F]DOPA with bioisosteric replacement of the C6 hydrogen with fluorine-18. [^18^F]FDG is an example of the latter, with replacement of the C2 hydroxyl group of glucose.

### Radiolabeling Peptides and Proteins With Fluorine-18

Given its attractive nuclear properties and wide availability, researchers have explored radiolabeling peptides and proteins with fluorine-18 for use in PET. Fluorine-18 is compatible with peptides and small to intermediate sized proteins (≤60 kDa) as they reach their molecular targets within times comparable to its moderate half-life of 110 min (Williams, 2012; Warnders et al., 2018). However, the complexity of these molecules makes radiolabeling them with fluorine-18 a challenging task.

For proteins specifically, there are numerous reactive centers and often the products are heterogeneously labeled. This heterogeneity may affect biodistribution, pharmacokinetics, affinity to the target, and solubility of the molecule, though these consequences are not always well understood (Krall et al., 2016). For example, Grierson et al. showed that the binding of annexin V to apoptotic cells is compromised when randomly radiolabeled with a fluorine-18 prosthetic group at multiple lysine residues vs. when site-selectively modified at cysteine (Grierson et al., 2004). Tait *et al.* also found that site-selective radiolabeling with technetium-99 lead to an increase in binding affinity of annexin V when compared to those randomly modified (Tait et al., 2006). In another study however, no difference was found between the random and site-selective modification of annexin V (Perreault et al., 2015). For these reasons, it is optimal to use site-selective labeling methods such as biorthogonal chemical modifications and unnatural amino acid incorporation to precisely incorporate fluorine-18 and avoid the issues associated with heterogeneously labeled products. Additionally, physiological conditions (an aqueous environment within a pH range of 5-8 and temperatures at or below 37°C) are generally required in the labeling step to prevent misfolding and maintain high specificity and affinity for their molecular targets.

#### Electrophilic vs. Nucleophilic Fluorine-18 Labeling

Radiolabeling with fluorine-18 can be conducted *via* either electrophilic or nucleophilic methods. For electrophilic fluorinations, fluorine-18 is produced by proton irradiation of [^18^O]O_2_ gas via the nuclear reaction ^18^O(p,n)^18^F. After irradiation the fluorine-18 produced adsorbs on the target walls and the addition of non-radioactive fluorine is required to recover the fluorine-18 *via* an isotopic exchange yielding [^18^F]F_2_ (Coenen, 2007). The extremely reactive [^18^F]F_2_ can then be used directly or, more commonly, converted to a less reactive fluorinating agent such as xenon difluoride ([^18^F]XeF_2_) or acetylhypofluorite ([^18^F]CH_3_COOF) (Yang et al., 2017). Electrophilic fluorination has played a critical role in the synthesis of imaging agents for PET. [^18^F]FDG for example, now the most widely used PET imaging agent, was first synthesized using electrophilic fluorination (Ehrenkaufer et al., 1984). However, the need to add non-radioactive fluorine during the synthesis of [^18^F]F_2_ results in lower specific activities (Alauddin, 2012). For peptides and proteins to be used in targeted imaging, high specific activities are essential as their molecular targets *in vivo* are readily saturated and often expressed in low densities (Richter and Wuest, 2014). Therefore, radiolabeling is most often conducted *via* nucleophilic fluorination with [^18^F]fluoride.

Nucleophilic [^18^F]fluoride ions are generated through irradiation of ‘heavy’ water ([^18^O]H_2_O) *via* the nuclear reaction ^18^O(p,n)^18^F. Fluoride ions are generally poorly nucleophilic in water (Hefter and McLay, 1988) however, rigorous drying procedures enables the use of [^18^F]fluoride as a nucleophile in radiolabeling experiments. This is commonly achieved by passing the solution through an ion-exchange cartridge to capture the [^18^F]fluoride ion which is then eluted as its alkali or tetrabutylammonium salt. To further activate the [^18^F]fluoride ion, the alkali salts can be chelated by cryptands. For example, after elution with potassium carbonate giving [^18^F]KF, the potassium cation can be complexed by Kryptofix 222 (Block et al., 1986; Coenen et al., 1986). In addition to the dry conditions, nucleophilic fluorinations are usually performed at elevated temperatures (50–100°C) to increase yields and specific activities as well as shorten reaction times.

In general, due to the low specific activities associated with electrophilic fluorinations, the low nucleophilicity of the fluoride ion in water and the high temperatures employed, proteins (and those peptides that also denature under these conditions) are most commonly indirectly radiolabeled with fluorine.

#### Indirect vs. Direct Radiolabeling of Peptides and Proteins

**FIGURE 1:**
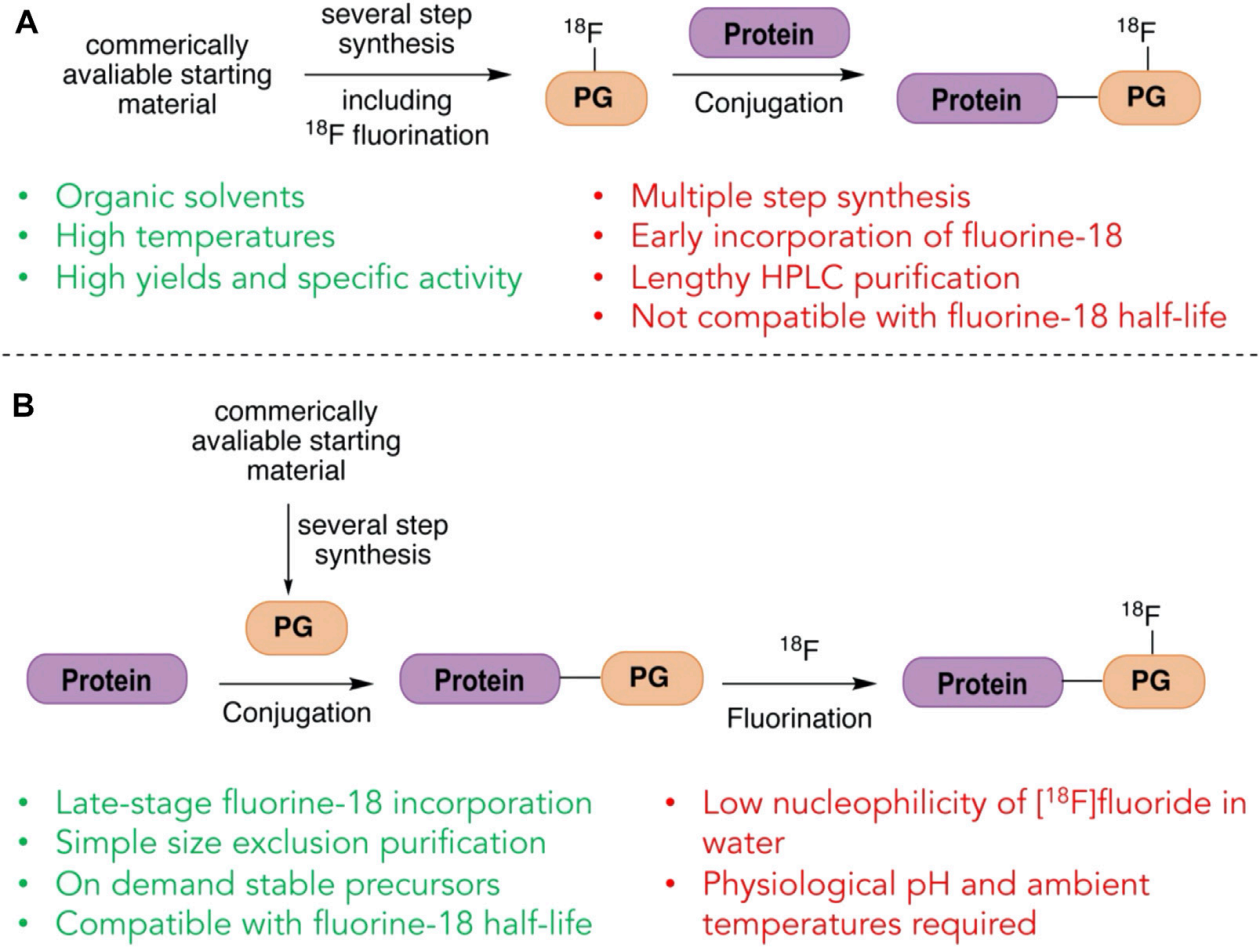
Indirect and direct strategies for labeling with fluorine-18. **(A)** In indirect labeling of proteins with fluorine-18, a prosthetic group (PG) is first labeled with fluorine-18 and purified before ligating to the protein. **(B)** In direct labeling of proteins with fluorine-18, the prosthetic group (PG) is attached first and the fluorine-18 labeling is done in the final step.

Indirect methods of radiolabeling involve the introduction of fluorine-18 through prosthetic groups; small compounds that can be radiolabeled, often at elevated temperatures in organic solvents, and then subsequently conjugated to a biomolecule under comparatively mild conditions (Figure 1A). The first indirect radiolabeling of a protein with fluorine-18 was reported in 1982 when Müller-Platz and co-workers used [^18^F]fluoroacetic acid as a prosthetic group to radiolabel urokinase. [^18^F]Fluoroacetic acid was prepared *via* nucleophilic fluorination of ethylbromoacetate followed by hydrolysis of the ester at reflux. [^18^F]Fluoroacetic acid was then conjugated to the free amino groups of urokinase under physiological conditions through amide coupling mediated by *N*-(3-dimethylaminopropyl)-*N*ʹ-ethylcarbodiimide (EDC) (Müller-Platz et al., 1982). An extensive number of prosthetic groups have since been used to radiolabeled proteins with fluorine-18 through indirect labeling methods (Kuhnast and Dollé, 2010; Krishnan et al., 2017; Schirrmacher et al., 2017). Notwithstanding the resourcefulness of indirect radiolabeling methods, these intrinsically multi-step syntheses are far from optimal, particularly for the short-lived fluorine-18 isotope.

Direct methods on the other hand, at least in principle, offer the flexibility of first incorporating a unique prosthetic group into proteins which can then be radiolabeled with fluorine-18 in a single, final step (Figure 1B). Such a method would allow the biomolecule to be made and potentially stored until needed, ready to be directly radiolabeled on-demand in the final step. This strategy also minimizes the number of chemical steps to which the fluorine-18 source is subjected, potentially increasing the radiochemical yield. It has however, been thought that in general proteins cannot be directly radiolabeled through traditional C-F bond formation as it requires conditions that may denature the protein (Ametamey et al., 2008) and researchers have turned to heteroatom-F bond formation as a possible alternative.

## Strategies for Directly Radiolabeling Peptides and Proteins With Through Heteroatom-And Metal-[^18^F]fluoride Bond Formation

Recently there has been a number of investigations looking into the radiolabeling of molecules with fluorine-18 through heteroatom-[^18^F]fluoride bond formation; specifically through the formation of [^18^F]fluoride bonds to boron, metals, silicon, phosphorus and sulfur. Similar to the C-F bond, heteroatom-F bonds are some of the strongest bonds with high bond dissociation energies [C-F: 536 kJ mol^−1^, B-F: 766 kJ mol^−1^, Al-F: 664 kJ mol^−1^, Si-F: 540 kJ mol^−1^, P-F: 439 kJ mol^−1^, S-F: 343 kJ mol^−1^ (Speight, 2017)] and have a relatively high stability. Si-F bonds for example often require activation before they will undergo reactions (Kameo and Sakai, 2015), as do B-F bonds (Kameo et al., 2020) and some S-F bonds (Pascali et al., 2017). This is an important feature to ensure that the radionuclide stays attached to the desired probe. In contrast, formation of many heteroatom-F bonds have lower activation energy barriers to their formation than that of C-F bonds. Kostikov et al. showed that the activation energy of [^19^F]/[^18^F] isotopic exchange at silicon is 65.6 kJ mol^−1^, lower than that of the fluorination at carbon *via* substitution of a tosylate (71.5 kJ mol^−1^) a common method used for the radiolabeling of compounds with [^18^F]fluoride (Kostikov et al., 2011). A lower activation energy can assist in mitigating the use of high temperature and the use of organic solvents detrimental to sensitive biomolecules. As an alternative to C-F bond formation, these strategies have rekindled the prospect of being able to directly radiolabel proteins with [^18^F]fluoride.

### Radiolabeling Through B-F Bond Formation

Boron was one of the first elements to be used as an inorganic fluoride acceptor for radiolabeling with [^18^F]fluoride. B-F bond formation for the radiolabeling of small molecules has been known since the 1960s (Askenasy et al., 1962; Entzian et al., 1964). However, until Ting *et al.* reported the radiolabeling of a biotinylated aryl boronic ester with [^18^F]fluoride in 2005 (Ting et al., 2005), boron’s use in the radiolabeling of biomolecules had not gained traction. In this report, the authors showed that the alkylboronic ester could readily be fluorinated in a single, rapid step with simple addition of [^18^F]fluoride, without the need for the rigorous and time-consuming drying generally employed. The fluorination worked well in aqueous solvents and yields approached 58 and 41% after an hour at pH 4.5 and 7.5 respectively. Additionally, the stability of the boron radiolabeled species was measured in blood and serum with no dissociation of [^18^F]fluoride observed up to 1 h. Thus, they suggested that [^18^F]trifluoroborates may be useful as imaging agents in PET. Since this report, a wide range of aryl and alkyltrifluoroborates have all been investigated for this purpose.

**FIGURE 2:**
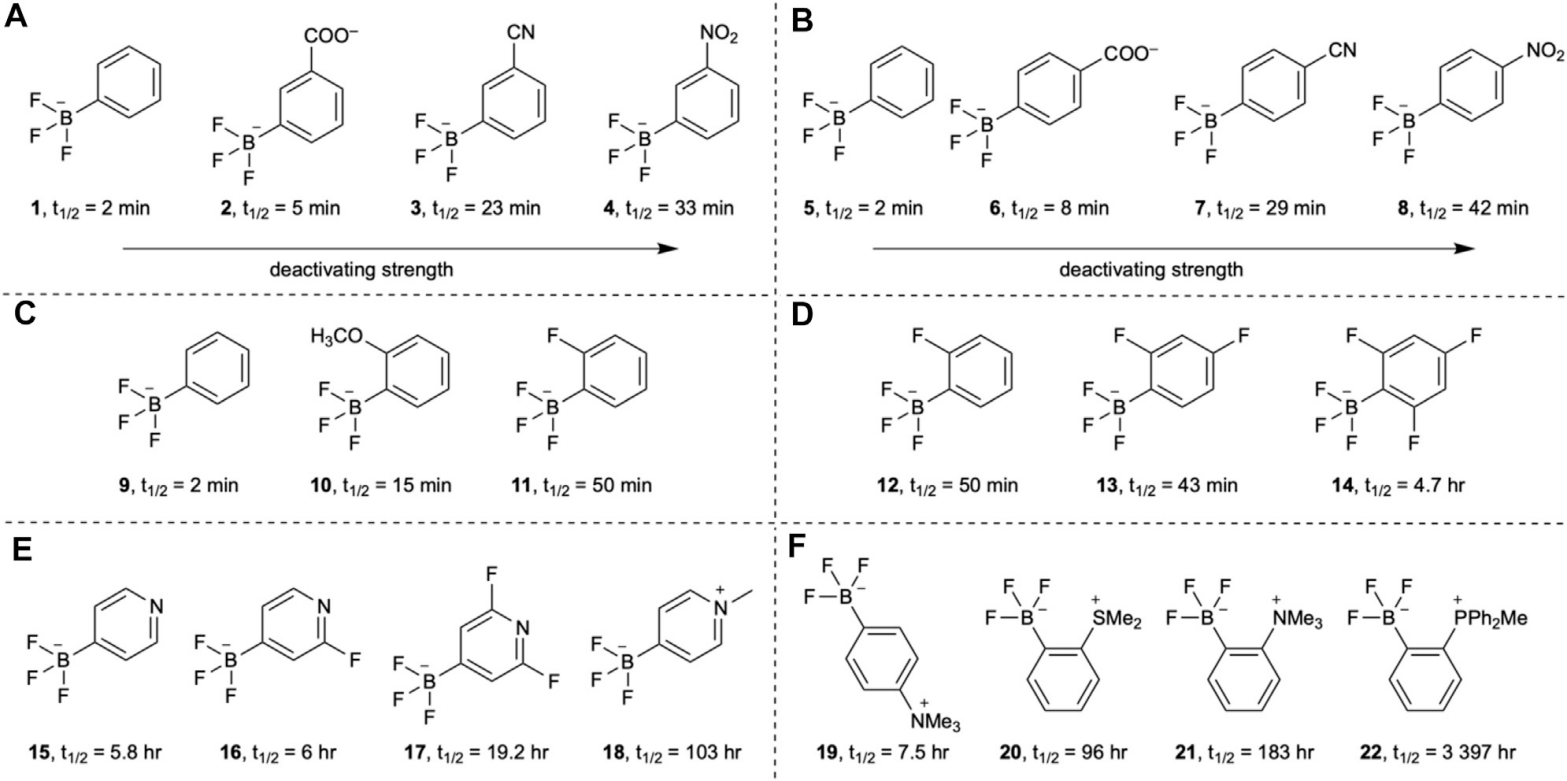
The effects of aromatic substitution on the hydrolytic half-life of the B-F bond of aryltrifluoroborates. The hydrolytic stability of the B-F bond is influenced by **(A)** *meta* substituents, **(B)** *para* substituents, **(C)** *ortho* substituents, **(D)** electron withdrawing fluorine substituents, **(E)** heteroaromatic effects, and **(F)** zwitterionic substituents.

**FIGURE 3:**
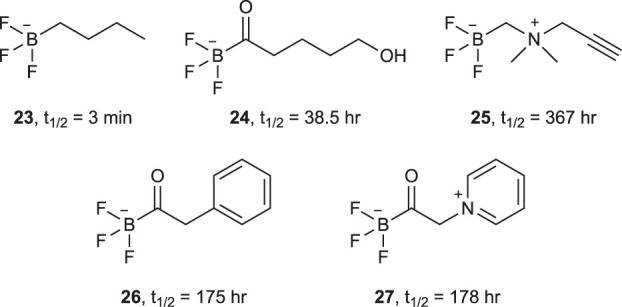
A selection of alkyl- and acyltrifluoroborates and their half-lives.

The hydrolytic stability of the B-F bond of aryltrifluoroborates is dependent on the substituents on the aryl group and their positions relative to the B-F bond. Perrin *et al.* showed that the B-F bond’s half-life can be increased through electron withdrawing substituents on the aromatic ring of the aryltrifluoroborate (Ting et al., 2008a; Ting et al., 2008b; Li et al., 2009) (Figures 2A–C). Substituents at *ortho* positions have a large effect on the half-life through both electronic nature and steric effects. An *ortho* methoxy substituent increases the half-life from 2 to 15 min and an *ortho* fluorine substituent to 50 min (Figure 2C). Interestingly, the addition of a *para* fluorine substituent to these molecules results in a decrease in the half-life but with fluorine substitution at both *ortho* positions and the *para* position the half-life reaches 4.7 h (Figure 2D). The introduction of endocyclic heteroatoms gives the largest increases in half-life, on the order of hours rather than minutes, which is more suitable for PET (Figure 2E). Gabbaï and co-workers further showed that zwitterionic aryltrifluoroborates also offer an even greater hydrolytic stability (Li et al., 2012). Substituted with cationic functionality at the *ortho* position, the close proximity to the trifluoroborate creates strong coulombic interactions thought to stabilize the B-F bond (Figure 2F). Perrin and co-workers also examined the rates of B-F bond hydrolysis of organotrifluoroborates using ^19^F NMR spectroscopy and determined their half-life (Liu et al., 2015a) (Figure 3). They established some predictive guidelines for the trifluoroborate stability, which are consistent with the effects observed in Figure 2. These effects can help design relevant probes for extension to [^18^F]fluoride labeling of the corresponding boronic acids or esters.

**FIGURE 4:**
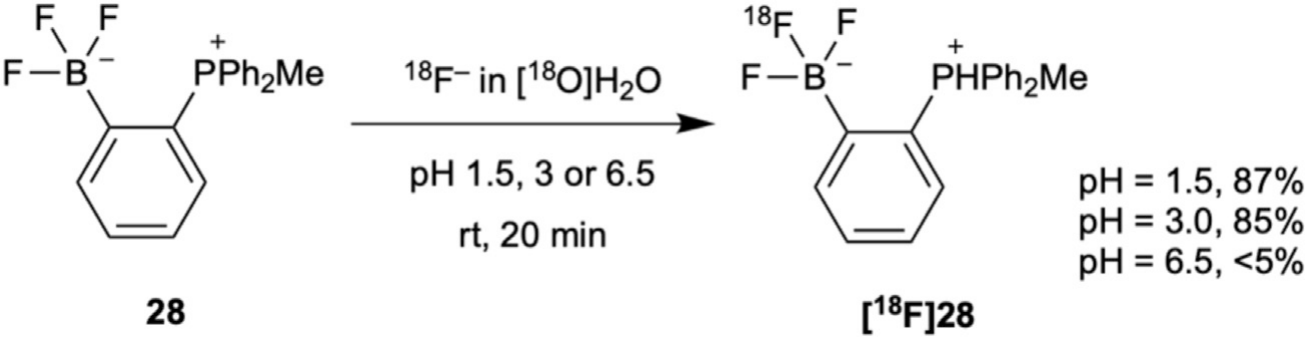
[^18^F]Trifluoroborates can be synthesized *via* ^19^F/^18^F isotopic exchange at low pH and room temperature in high yields in only 20 min.

[^18^F]Trifluoroborates, and their non-radioactive counterparts, can be readily synthesized from their corresponding boronic acids or esters under acidic conditions. Fluorination is optimal at pH 2-3, when the hydroxide or alkoxy substituents can be protonated. These acidic conditions can be generated by the addition of the carrier KHF_2_ and/or hydrochloric acid. [^18^F]Trifluoroborates can also be synthesized through ^19^F/^18^F isotopic exchange. Inspired by the success of ^19^F/^18^F isotopic exchange in fluorosilanes discussed in the sections that follow, Gabbaï *et al.* showed that trifluoroborates could also be radiolabeled *via* ^19^F/^18^F isotopic exchange simply by reacting a solution of [^18^F]fluoride in target water and the trifluoroborate in acetonitrile (<15% total volume) (Li et al., 2012) (Figure 4). The reaction proceeded efficiently at room temperature in only 20 min at a pH of 1.5–3.0. At higher pH, however, reduced yields were obtained.

The rapid exchange reaction shown in Figure 4 and related fluorine labeling at boron have been applied to the radiolabeling of a number of inhibitors, probes, and biomolecules including biotin (Harwig et al., 2008), rhodamine (Liu et al., 2012), metalloproteinase inhibitor marimastat (auf dem Keller et al., 2010; Li et al., 2011), histone deacetylase inhibitor panobinostat (Kommidi et al., 2018), bombesin (Li et al., 2013a), cycloarginylglycylaspartic acid (RDG) (Li et al., 2013b), boramino acids transportation markers (Liu et al., 2015b; Liu et al., 2015c) and a smart furin-controlled self-assembly tracer (Zhao et al., 2020). These examples reflect the efficiency and versatility of this labeling strategy.

### Radiolabeling Through Metal Complexes

Metal complexes have also proven useful as fluoride acceptors for the radiolabeling of peptides and proteins for PET imaging. Envisioned as a method complementary to radiometal labeling (McBride et al., 2009), this strategy makes use of [^18^F]fluorine-metal complexes that can be chelated to ligands incorporated into a peptide or protein. Since the first reported use of metal fluorine complexes for radiolabeling with [^18^F]fluoride in 2009 (McBride et al., 2009) this field of research has moved forward rapidly with group 13 metals having particular success.

**FIGURE 5:**
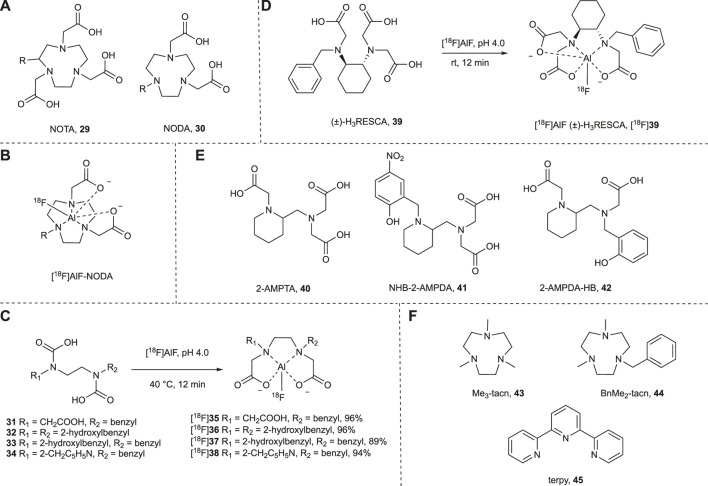
**(A)** NOTA- and NODA-derived chelating agents. The NOTA derivative offers six donor atoms which compete with the [^18^F]fluoride ion in coordination with the aluminum ion. In comparison the NODA derivative has only five donor atoms leaving a coordination site free for the [^18^F]fluoride ion. When R = H **29** = NOTA and **30** = NODA. **(B)** [^18^F]AlF coordinated to the NODA-derived complex. R can also be the site of ligation to a peptide for all compounds shown. **(C)** Acyclic chelators allow for complexation with [^18^F]AlF in aqueous solvents at 40°C, pH 4.0 in 12 min. **(D)** Complexation of [^18^F]AlF to (±)-H_3_RESCA occurs at room temperature, pH 4.0 in only 12 min in aqueous solvents. **(E)** Acyclic chelators that allow for complexation with [^18^F]AlF in aqueous solvents at 40°C, in 12 min at high pH (4.5–6.5). **(F)** A selection of chelators with three nitrogen donors that have been explored by Reid and co-workers for the complexation of fluorine to aluminum, gallium, indium, scandium, yttrium, lanthanum, lutetium, chromium, manganese, iron and cobalt.

McBride *et al.* were the first to report the use of aluminum fluorine metal complexes for the radiolabeling of compounds with [^18^F]fluoride through chelation (McBride et al., 2009). In this initial study, the ability of [^18^F]fluorine-metal complex to bind to a diethylenetriaminopentaacetic acid (DTPA) hapten-peptide was investigated. Of the six metals tested (aluminum, gallium, indium, zirconium, lutetium and yttrium) the aluminum complex bound to the DTPA-hapten-peptide with the greatest affinity, but unfortunately none were stable in water. As aluminum fluoride complexes are known to be stable in water, the authors attributed the low stability to the weak binding of the metal to the DTPA chelate. Thus, they screened a number of other chelates and assessed their stability. In all cases, yields were lower than what could be achieved with DTPA and only when [^18^F]AlF was bound to a 1,4,7-triazacyclononane-1,4,7-triacetic acid (NOTA) derivative, did it exhibit sufficient stability in serum and *in vivo*. It has since been shown that greater labeling yields can be achieved with the pentadentate NOTA derivative, NODA, without a loss in stability (Figure 5A) ((McBride et al., 2010; D’Souza et al., 2011; Shetty et al., 2011). Only coordinating with the aluminum ion at five donor atoms instead of six leaves free a final coordination site for the [^18^F]fluoride ion (Figure 5B).

The [^18^F]AlF metal complex can be synthesized from aluminum trichloride in water at room temperature. This is optimal at pH 4.0 and thus is generally performed in sodium acetate buffer. Binding of the complex with chelating ligands is also optimal at pH 4.0, typically at temperatures of 100°C. A one-pot method whereby aluminum trichloride, [^18^F]fluoride and the chelate are all added together has been extensively used for the radiolabeling of peptides with fluorine-18 (for recent reviews see Kumar and Ghosh, 2018; Fersing et al., 2019). This approach with heat sensitive proteins has been limited to indirect radiolabeling of a small molecule with subsequent conjugation of the radiolabeled chelate to the protein (McBride et al., 2012; Lütje et al., 2014; Da Pieve et al., 2016; Lu et al., 2017; Basuli et al., 2018; Zhou et al., 2018; van der Veen et al., 2020). There have, however, been some efforts made toward reducing the temperature and increasing the pH such that radiolabeling through aluminum complexes may be suitable for the direct radiolabeling of heat and acid sensitive proteins.

For example, Huynh *et al.* were able to reduce the temperature required for complexation of [^18^F]AlF to NODA chelators by increasing the pH to 5.5. To obtain sufficient yields high levels (>40%) of ethanol were required (Huynh et al., 2019). Nevertheless, they were able to radiolabel the monoclonal antibody trastuzumab at 30°C in 15 min with a 45% radiochemical yield and it showed a greater uptake in HER2 positive cells (9.1 ± 1%) than it did HER2 negative cells (0.8 ± 0.04%), suggesting the high levels of ethanol did not affect its affinity for its molecular target.

In 2016 Cleeren *et al.* synthesized new chelators that were able to bind with [^18^F]AlF at 40°C without any organic co-solvent and at the optimal pH of 4.5 (Cleeren et al., 2016). These chelators were designed to be acyclic rather than macrocyclic to reduce the activation energy of the chelation. In order to maintain the stability that may have been lost by this change, they also replaced one nitrogen donor with an oxygen donor, as it is a more effective aluminum chelator. [^18^F]AlF complexed to four of the eight chelators tested in exceptional yields at 40°C in 12 min (Figure 5C). At room temperature, **31** could be radiolabeled in a >90% RCY but 40°C was needed for **32**, **33** and **34**. **[** ^**18**^ **F]37** showed sufficient stability *in vitro* (rat serum, 37°C) and *in vivo* (healthy mice). Therefore, as a proof of concept, **33** was conjugated to a urea-based prostate-specific membrane antigen (PSMA) inhibitor. Radiolabeling with [^18^F]AlF at pH 4.5, 40°C for 12 min resulted in a 25% RCY. Preliminary studies into the stability *in vivo* were promising but require further evaluation. Cleeren *et al.* have since reported a new restrained chelator, (±)-H_3_RESCA (**39)**, that can be complexed to [^18^F]AlF at room temperature in 12 min at pH 4.5 (Musthakahmed et al., 2016) (Figure 5D) and a protocol for using it to radiolabel proteins (Cleeren et al., 2018). This procedure has successfully been used to directly radiolabel human serum albumin (HAS), Kupffer cell marker CRIg, and a HER2 targeting affibody with fluorine-18 (Cleeren et al., 2017).

One downside to the method developed by Cleeren and co-workers for the direct radiolabeling with [^18^F]fluoride is that the reaction is performed at pH 4-5 and may not be suitable for acid sensitive proteins. As previously discussed, Huynh *et al.* was able to achieve complexation at pH 5.5 and room temperature using high concentrations of ethanol (Huynh et al., 2019). Russelli *et al.* has recently reported three chelators that can be radiolabeled at higher pH using Cleeren’s method in good yields (Figure 5E) (Russelli et al., 2020). At pH 4.0 only **40** could be efficiently labeled but at pH 5.0 all three were labeled efficiently (81, 69 and 52% for **40**, **41** and **42** respectively). Even at pH 6.5 radiochemical yields reached *ca.* 50% for all three chelators. **42** showed the greatest stability *in vitro* and was further studied *in vivo*. Preliminary results indicate that it has an adequate hydrolytic stability with a lower accumulation of free [^18^F]fluorine in bone than observed for previously reported [^18^F]AlF complexes (Cleeren et al., 2016).

Alongside aluminum, a whole suite of fluorine-metal complexes have been explored for fluorine-18 radiolabeling purposes. Reid and co-workers explored Cl/F halide exchange with aluminum, gallium and indium trichlorides complexed to the macrocyclic chelators **43** and **44** (Figure 5F) (Bhalla et al., 2014). All the fluorine-19 complexes could be synthesized from their corresponding trichlorides at room temperature but organic solvents (100% for In, 70% for Al and Ga) were used and only GaCl_3_(BnMe_2_-tacn) was radiolabeled. In a 1:1 water:acetonitrile mixture at room temperature, a 30% RCY was achieved in 1 h using carrier added [^18^F]KF. Comparatively, when GaCl was complexed to a NODA chelator, the same results could be achieved in only 30 min at room temperature without the addition of an organic co-solvent (Bhalla et al., 2015). [^18^F]GaF_3_(BnMe_2_-tacn) has also be synthesized through ^19^F/^18^F isotopic exchange, however, it was performed in 25% water and required heating to 80°C (Monzittu et al., 2018). Reid *et al.* have also reported the radiolabeling of AlCl_3_(BnMe_2_-tacn) using McBride’s method (pH 4.0, 100°C) with carrier added [^18^F]KF that lead to a RCY of 24% in 60–90 min (Levason et al., 2017).

Reid and co-workers also reported attempts of Cl/F halide exchange of the trichloride **43**, **44** and **45** complexes (Figure 5F) with the metals scandium, yttrium, lanthanum and lutetium, though only the scandium complexes could be synthesized using this method and it required anhydrous conditions (Curnock et al., 2018). Most recently they have investigated chromium, manganese, iron and cobalt in their corresponding trichloride complexes (**43**, **44** and **45**) (Blower et al., 2019). The cobalt and manganese complexes were found to be unstable in water while the halide exchange for the chromium complex did not go to completion even after 24 h at reflux in acetonitrile. Iron on the other hand underwent exchange in aqueous acetonitrile at room temperature in just 30 min using 4 mol equivalent of potassium fluoride when complexed with **44**. This complex could be radiolabeled in a 6% yield with aqueous [^18^F]fluoride in a 1:4 water:acetonitrile mixture at 80°C in 10 min.

### Radiolabeling Through Si-F Bond Formation

**FIGURE 6:**
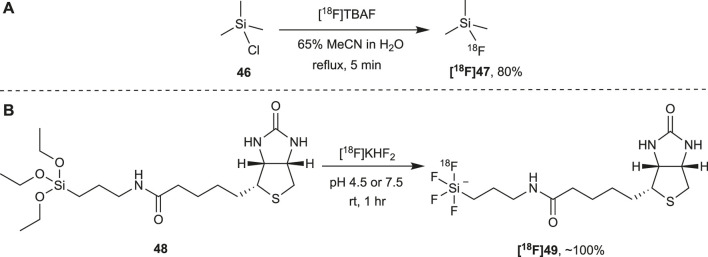
**(A)** The first reported direct nucleophilic radiolabeling of a molecule with [^18^F]fluoride at silicon under aqueous conditions involved the fluorination of silyl chloride **46** with [^18^F]TBAF. **(B)** The biotin derivative **48** can be directly radiolabeled with [^18^F]fluoride at room temperature over a broad pH range and within an hour in almost quantitative yields. Carrier KHF_2_ is added to obtain a Si:F ratio of 1:4. Theoretically, **49** could be radiolabeled with [^18^F]fluoride a total of four times which would increase the specific activity substantially.

The first report of Si-F bond formation for radiolabeling was in 1985 when Rosenthal and co-workers successfully prepared [^18^F]fluorotrimethylsilane (**[** ^**18**^ **F]47**) by reacting **46** with [^18^F]tetrabutylammonium fluoride ([^18^F]TBAF) (Figure 6A) (Rosenthal et al., 1985). In contrast to C-F bond formation this reaction proceeded even in the presence of water and **[** ^**18**^ **F]47** was produced in an 80% yield (decay corrected) when performed in a 65% acetonitrile aqueous solution. It would, however, be another 20 years before Si-F bond formation was revisited for the radiolabeling of biomolecules with fluorine-18 when in 2005 Ting *et al.* reported the radiolabeling of the triethoxysilane biotin derivative **48** with [^18^F]fluoride through Si-F bond formation (Figure 6B) (Ting et al., 2005). In this reaction, the [^18^F]fluoride anion was used directly without conversion to another fluorinating agent and simply added to an aqueous solution of **48**. This single step fluorination resulted in the rapid formation of alkyltetrafluorosilicate **[** ^**18**^ **F]49** with yields approaching 100% in buffered aqueous media at both pH 4.5 and 7.5 in less than an hour at room temperature.

These two foundational studies by Rosenthal *et al.* and Ting *et al.* revealed that Si-F bond formation may be a promising alternative to C-F bond formation for the direct radiolabeling of proteins with [^18^F]fluoride. In both examples, the Si-F bond formation was high yielding and achieved in time frames suitable to the half-life of fluorine-18, at room temperature and in aqueous solutions. To be useful, however, the Si-F bond needs to be stable *in vivo*. This is of utmost importance in PET as the probe cannot be imaged if the [^18^F]fluoride is cleaved from its structure. Unfortunately, in both cases described above a low hydrolytic stability of the Si-F bond was observed. *In vivo* [^18^F]fluorotrimethylsilane **([^18^F]47)** rapidly hydrolyzed (t_1/2_ < 1.5 min) and analysis *in vitro* found that the rate of hydrolysis increased at higher pH (Rosenthal et al., 1985). The alkyltetrafluorosilicate **([^18^F]49)** was also found to be susceptible to hydrolysis *in vitro* with hydrolysis observed within an hour when in a buffered aqueous solution at pH 7.5 (Ting et al., 2005).

Despite the strength of the Si-F bond, in dilute aqueous environments, the hydrolysis of Si-F bond is both thermodynamically and kinetically favored (Ting et al., 2005). Nevertheless, when fluorine-18 decays, it decays to oxygen-18 resulting in the same silanol product that results from hydrolysis. Therefore, the Si-F bond need only be stable for long enough that it outlives the fluorine-18 radionuclide. To alleviate the rate at which the Si-F bond hydrolyses Rosenthal *et al.* suggested incorporating more hindered substituents on the silicon atom (Rosenthal et al., 1985). Presumably, the steric and inductive effects of the substituents would reduce the ease at which the pentacoordinate hydrolysis transition state forms, as is the case for organosilanes.

**FIGURE 7:**
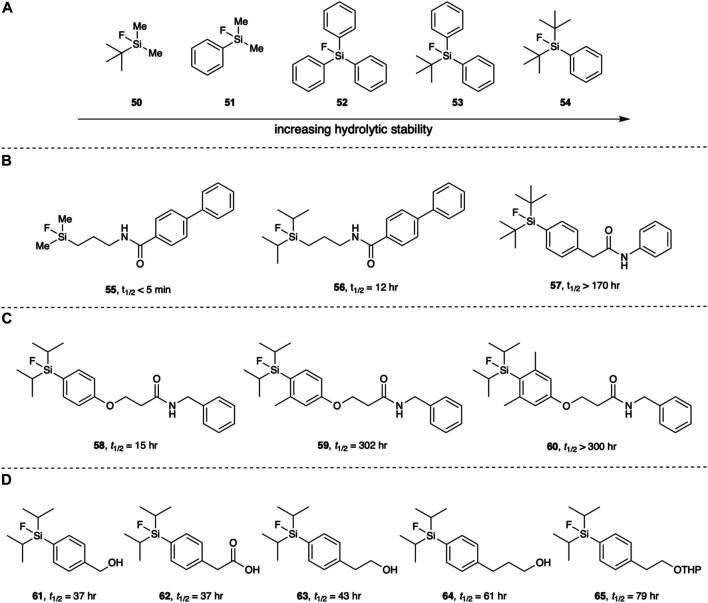
**(A)** The hydrolytic stability of the Si-F bond increases with an increase in steric hinderance around the silicon atom. **(B)** The hydrolytic half-life is affected by the silicon substituents. **(C)** The addition of methyl groups *ortho* to the aryl silicon increases hydrolytic stability. **(D)** *Para* substituents relative to the silicon atom also impart a subtle effect on the hydrolytic stability of the Si-F bond. All stated half-lives were measured in a 2:1 solution of MeCN:aqueous buffer (pH 7).

Independently, Choudhry and Schirrmacher showed that indeed, with an increase in steric congestion at silicon comes an increase in the hydrolytic stability of the Si-F bond. Choudhry reported that of the four compounds they tested (**50–53**) only **53** showed satisfactory stability *in vitro* with almost 100% stability observed after 5 h at 45°C in water and in PBS. Schirramcher *et al.* also tested **54** and observed the hydrolytic stability of the Si-F bond *in vivo*. It was found that while **53** did have reasonable stability in human serum at 37°C and pH 7.4–7.6 *in vivo*, hydrolysis occurs with the characteristic uptake of radioactivity in bones, indicating the presence of free [^18^F]fluoride. They found that the more sterically hindered **54** was stable in human serum and showed a limited uptake of radioactivity in bone, *in vivo,* 50 min post injection (Figure 7A) (Choudhry et al., 2006; Schirrmacher et al., 2006).

Ametamey and co-workers showed the diisopropyl substituents also increase the hydrolytic stability. They found Si-F bonds with diisopropyl substituents to be more stable against hydrolysis than methyl substituents but less than *tert*-butyl substituents (Figure 7B) (Mu et al., 2008). In a follow up paper, Ametamey and co-workers further explored the hydrolytic stability of the Si-F bond determining the hydrolytic half-lives of fifteen fluorosilanes (Höhne et al., 2009). Their results corroborated previous studies and assumptions that bulkier substituents on the silicon leads to an increased hydrolytic stability of the Si-F bond. The addition of methyl groups at the *ortho* positions of the phenyl ring were also found to give a significant increase in hydrolytic half-life (Figure 7C). Interestingly, the functionality at the *para* position of the phenyl ring had a subtle influence on the hydrolytic stability (Figure 7D). This suggests that inductive effects, not just steric effects, can modulate the hydrolytic stability of Si-F bonds. Outside of this study however, the role inductive effects play in the hydrolytic stability of Si-F bonds have not been explored. Nevertheless, those arylfluorosilanes substituted with either the diisopropyl or di-*tert*-butyl groups displayed a satisfactory hydrolytic half-life to be useful PET imaging agents.

[^18^F]arylfluorosilanes can be synthesized from their corresponding silanes, silanols and fluorosilanes. They have also been synthesized from chlorosilanes and alkoxysilanes, but both undergo rapid hydrolysis when in aqueous solutions (Szabó et al., 2014; Issa et al., 2019). Thus, they have a very limited applicability for the direct radiolabeling of proteins. On the other hand, silanes are relatively stable in neutral and slightly acid media. Fluorosilanes with sufficiently bulky substituents are also reasonably stable in water as shown in Figure 7, and silanols are also stable in water. It is therefore reasonable to explore these groups as sites for fluorination or fluoride exchange.

**FIGURE 8:**
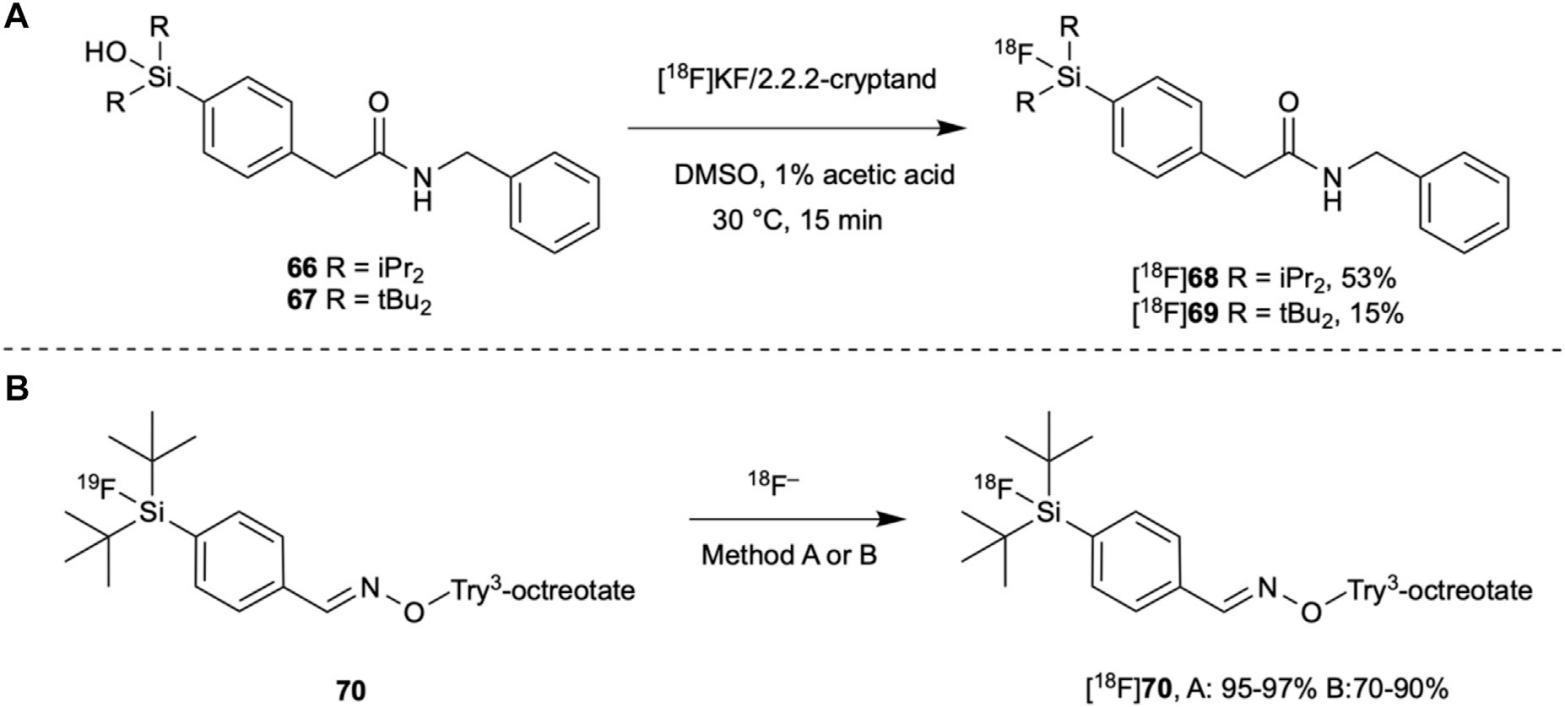
(**A**) Silanols can be radiolabeled with [^18^F]fluoride at room temperature in organic solvents though bulker substituents on the silicon atom result in a lower yield. (**B**) Try^3^-octreotate has been radiolabeled with [^18^F]fluoride in both organic and aqueous solvents. Method A: [^18^F]KF/Kryptofix 222, MeCN, rt, 10–15 min. Method B: [^18^F]F^−^/[^18^O]H_2_O, MeCN (15–20% total volume), 95 °C, 30 min.

The nucleophilic fluorination of di-isopropyl and di-*tert*-butyl aryl silanols and silanes was explored by Ametamey and co-workers (Mu et al., 2008). Fluorinations were performed in DMSO using the azeotropically dried ^18^F[KF]/Kryptofix 222 complex. The silane derivatives underwent fluorination more readily with lower conversions observed for the silanols. Conversion could be increased by the addition of acetic acid, possibly by protonating the hydroxyl group of the silanol, thereby creating a better leaving group. While they did not test the radiolabeling experiments in an aqueous solution, it is likely that slightly acidic conditions would be required to obtain fluorosilanes from silanols in reasonable yields (Figure 8A). This study also brought to light a trade-off between the ease of fluorination and an increased hydrolytic stability. For example, **[** ^**18**^ **F]68** was obtained in a 53% yield from its silanol precursor **66** when fluorinated at 30°C for 15 min with the addition of acetic acid. In comparison, the bulkier **[** ^**18**^ **F]69** was obtained from its silanol precursor **67** in a 15% yield under the same reaction conditions. These yields were further increased at 65°C to 90 and 23% yields, respectively (Mu et al., 2008). In a later study, this trend was also observed when fluorinating silanes (Höhne et al., 2009).

Despite the *tert*-butyl groups slowing fluorination, their steric bulk still offers a greater hydrolytic stability than less bulky substituents and they can be synthesized in good yields when fluorinated *via* ^19^F/^18^F isotopic exchange. In fact, ^19^F/^18^F isotopic exchange has been an increasingly common method for radiolabeling biomolecules with [^18^F]fluoride through Si-F bond formation. It had previously been avoided as a radiolabeling method due to low yields and specific activities due to the reversible nature of the reaction. However, when conditions can be modified to obtain higher yields and specific activities, the purification step is simplified since it is a degenerate reaction, changing only the isotope of the fluorine. Simplifying the purification step can therefore save valuable time in the synthesis of fluorine-18 tagged imaging agents.

Schirrmacher and co-workers were the first to show that ^19^F/^18^F isotopic exchange was applicable to Si-F bond formation. Remarkably, when radiolabeling di-*tert*-butylphenyl [^19^F]fluorosilane, yields of 80–95% were achieved in just 10–15 min at room temperature when using the azeotropically dried [^18^F]KF/Kryptofix 222 complex in acetonitrile (Schirrmacher et al., 2006). Furthermore, when conjugated to Tyr^3^-octreotate (**70**), fluorination yields of 95–97% were obtained (Figure 8B). They also tried to radiolabel **70** in a 15% acetonitrile aqueous solution with [^18^F]fluoride in target water directly after irradiation. Unfortunately, at room temperature for 15 min this only gave **[** ^**18**^ **F]70** in a 5% radiochemical yield. However, when the temperature was increased to 95°C and the reaction time doubled, the yields increased to 70–90%.

**FIGURE 9:**
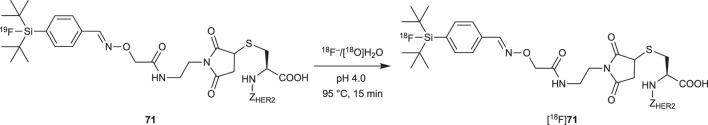
^19^F/^18^F isotopic exchange has been used to radiolabel the HER2 binding affibody under aqueous condition. This is the only example of the direct and aqueous fluorination of a large biomolecule in the chemical literature.

Requiring such high temperatures to obtain sufficient yields ^19^F/^18^F isotopic exchange has a limited applicability to the direct radiolabeling of proteins. Nevertheless, Glaser *et al.* has used this method to synthesize a fluorine-18 labeled human epidermal growth factor receptor (HER2) specific binding affibody (Glaser et al., 2013). Conjugating di-*tert*-butylphenyl [^19^F]fluorosilane to the affibody using maleimide chemistry followed by isotopic exchange with [^18^F]fluoride in target water yielded the affibody **[** ^**18**^ **F]71** in a 38% radiochemical yield in just 15 min at 95°C under 100% aqueous conditions, pH 4.0 (Figure 9). Though there was a limited difference in the retention of the radiolabeled affibody in high-HER2-expressing and low-HER2-expressing tumors, the binding affinity remained within a sub-nanomolar range. This indicates that the high temperatures used did not affect its specificity nor its affinity for its molecular target. To the best of our knowledge, this example reported by Glaser *et al.* represents the only example of the direct radiolabeling of a protein with [^18^F]fluoride in the chemical literature. This is a significant achievement because this strategy allows the use of the radioactive [^18^F]fluoride directly as it is produced, rather than requiring multiple manipulations of the PET active isotope.

Other than the single example in Figure 9, radiolabeling *via* this method has only been used for the indirect radiolabeling of proteins with [^18^F]fluoride (Iovkova et al., 2009; Rosa-Neto et al., 2009; Wängler et al., 2009; Kostikov et al., 2012; Koudih et al., 2014). Few other methods of aqueous Si-F radiolabeling methods have been described. Katzenellenbogen and co-workers reported the direct radiolabeling of a silylacetate (Kim et al., 2013) and Fouquet *et al.* has reported direct radiolabeling of silyl *N*-methyl-imidazoles (Tisseraud et al., 2018). Aqueous solutions of [^18^F]fluoride were used in both cases but ultimately, the reactions were performed in organic solvents (THF or acetonitrile) and at high temperatures (100–110°C). Recently, Scroggie and co-workers revisited direct fluorination of silanols in aqueous conditions. Useful conversions and rates of fluorination on silanol-labeled amino acids in the presence of water portends to future exploration of this chemistry on peptides and proteins, for ultimate use in direct fluorination of proteins (Scroggie et al., 2018).

### Radiolabeling Through P-F Bond Formation

**FIGURE 10:**
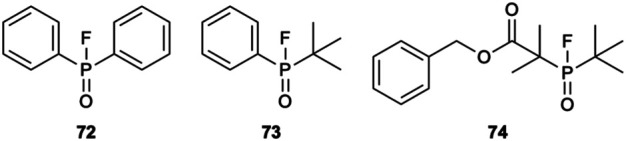
**72–74** have been radiolabeled through ^19^F/^18^F isotopic exchange. **74** offers the greatest hydrolytic stability and can be radiolabeled in a 50% radiochemical yield in a 95% aqueous solution at room temperature within 5–15 min.

Recently, the radiolabeling of proteins through phosphorus fluorine bond formation was reported (Hong et al., 2019). As with the other inorganic elements we have discussed, phosphorus readily forms bonds with fluorine. Hong *et al.* studied the effects of steric hinderance on radiolabeling yields and hydrolytic stability of the P-F bond in the small organophosphorous compounds **72–74** (Figure 10). When using ^19^F/^18^F isotopic exchange, rapid ^18^F-labelling occurring at room temperature within 5–15 min even with bulky *tert*-butyl substituents on the phosphorous atom. Notably, in a 95% aqueous solution (5% DMSO added for solubility of **74**) 50% radiochemical yields were achieved. Again, the use of two *tert*-butyl substituents lead to an improved hydrolytic stability and **[** ^**18**^ **F]74** was found to be 100% stable *in vivo* 120 min post injection into healthy mice. Using the tetrafluorophenyl ester of **74,** the organophosphine was conjugated to human serum albumin. No conditions for the procedure for the radiolabeling of the protein conjugate were detailed but a radiochemical yield of >5% was reported.

### Radiolabeling Through S-F Bond Formation

**FIGURE 11:**
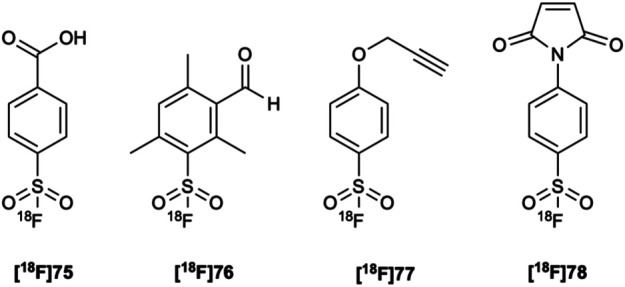
**[** ^**18**^ **F]75–78** can be synthesized in high radiochemical yields from their corresponding sulfonyl chlorides with [^18^F]CsF in 50% aqueous solutions at room temperature in only 15 min.

Inkster *et al.* synthesized **[** ^**18**^ **F]75**–**78** (Figure 11) from their corresponding sulfonyl chlorides through nucleophilic fluorination with [^18^F]CsF in 1:1 solutions of aqueous Cs_2_CO_3_ and organic solvents at room temperature in 15 min (Inkster et al., 2012). **[** ^**18**^ **F]76** was also synthesized with [^18^F]CsF in 100% aqueous Cs_2_CO_3_ however, variable yields were obtained (6–19%). The authors attributed this to the limited solubility of **76** in water and that the reaction was analyzed directly. When DMSO was added immediately before analysis, excellent radiochemical yields of 80% were recorded. Likely, these high yields are somewhat driven by the precipitation of the product from solution so extension to protein substrates would require further evaluation.

**FIGURE 12:**
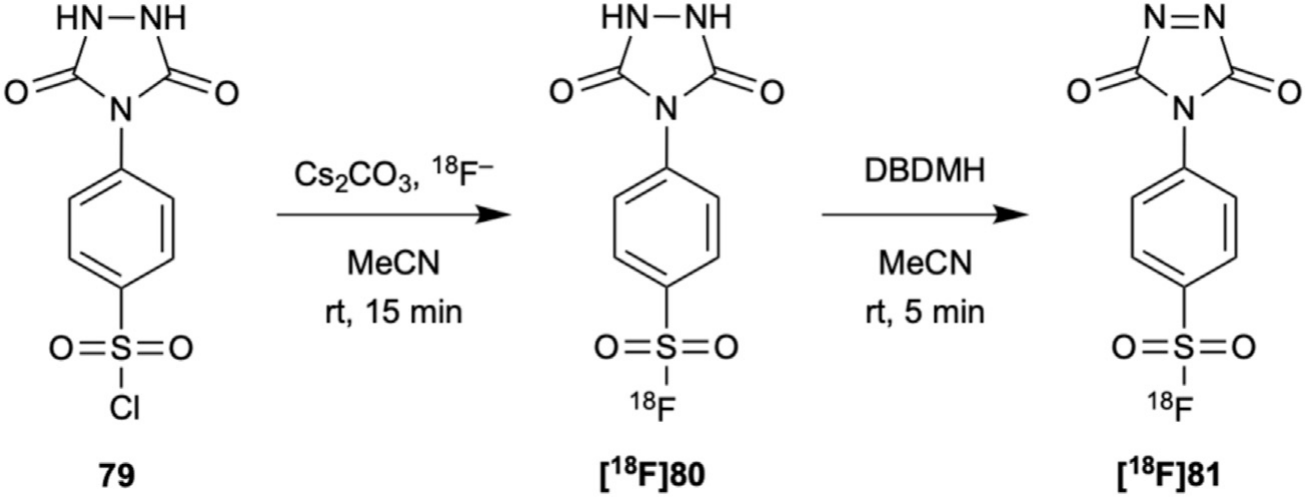
**[** ^**18**^ **F]81** was synthesized from its corresponding sulfonyl chloride **79** using [^18^F]fluoride in a cesium carbonate aqueous solution followed by oxidation with 1,3-dibromo-5,5-dimethylhydantoin (DBDMH). **[** ^**18**^ **F]81** reacts with tyrosine under basic (pH 9–10) conditions at room temperature and could be potentially used for the indirect radiolabeling of proteins at this residue.

Despite the ability to radiolabel arylsulfonyl chlorides in good yields under aqueous conditions and at room temperature, they unfortunately are susceptible to hydrolysis. Thus, they may have limited applicability to the direct radiolabeling of proteins. The method of radiolabeling an arylsulfonyl chloride described above has however, been used in the synthesis of **[** ^**18**^ **F]81** (Figure 12), a potential prosthetic group for the indirect radiolabeling of proteins at tyrosine (Al-Momani et al., 2015).

As with the other fluoride bonds we have discussed, S-F bonds also undergo hydrolysis. Inkster *et al.* found **76** and **78** to be hydrolytically stable at pH 7.2 in a 150 mM PBS solution with 10% DMSO over 2.5 h. **77** showed some hydrolysis (90% remaining) while **75** showed complete hydrolysis. Interestingly, this indicated that not only does steric hinderance protect the S-F bond from hydrolysis but that electronic affects may play a significant role (Inkster et al., 2012). However, Matesic and co-workers later proved that this is not the case and that while electron-donating groups may help stabilize the S-F bond, the more significant factor is steric hindrance (Matesic et al., 2013). Nevertheless, at the time given the excellent stability of **[** ^**18**^ **F]76,** it was used to indirectly label a bombesin peptide fragment through oxime formation in DMSO. Under identical conditions used for **[** ^**18**^ **F]75–78**, the radiolabeled peptide fragment was found to be hydrolytically stable, however, in mouse serum defluorination was observed within 15 min.

**FIGURE 13:**
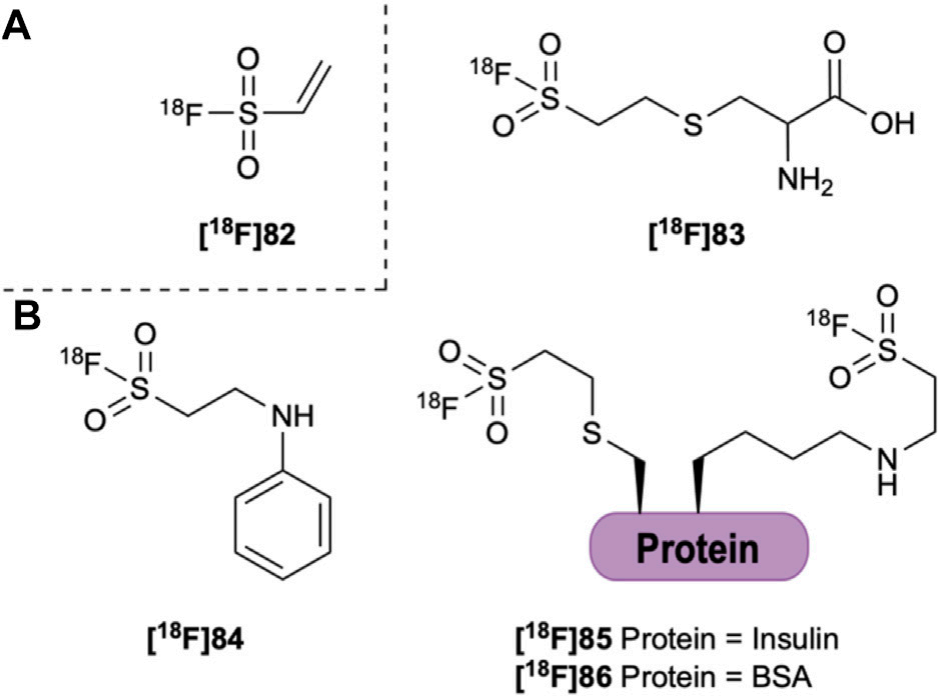
**(A)** Ethenesulfonyl [^18^F]fluoride (**[** ^**18**^ **F]82**). **(B) [** ^**18**^ **F]82** can be conjugated to aniline (**[** ^**18**^ **F]84**) and the amine of several amino acids, the thiol of cysteine (**[** ^**18**^ **F]83**) and to insulin (**[** ^**18**^ **F]85**) and BSA (**[** ^**18**^ **F]86**).

The Michael acceptor ethenesulfonyl [^18^F]fluoride (**[** ^**18**^ **F]82**, Figure 13A) has also been investigated as a prosthetic group, but shows a low stability in rat serum at 37°C (Zhang et al., 2018). The stability of the S-F bond was found to be highly dependent on the conjugate. After 15 min, the purity of the aniline adduct **[** ^**18**^ **F]84** had reduced to 70% while complete degradation of the cysteine adduct **[** ^**18**^ **F]83** was observed. **[** ^**18**^ **F]82** was also used to indirectly radiolabel insulin and bovine serum albumin (BSA). After only 15 min the purity of [^18^F]fluoroinsulin (**[** ^**18**^ **F]85**) had reduced to 13% while [^18^F]fluoroBSA (**[** ^**18**^ **F]86**) had completely degraded (Zhang et al., 2018). This suggests that as with B-F and Si-F bonds, the nature of distant functionalities also affect the hydrolytic stability of S-F bonds. The synthesis of di-*tert*-butyl analogues has been suggested as a way to increase the hydrolytic stability of the S-F bond. Preliminary studies investigating the synthesis of sterically hindered S-F compounds, however, have not overcome these challenges (Pascali et al., 2017).

**FIGURE 14:**
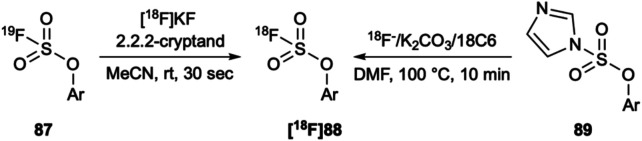
**(A)** Aryl [^18^F]fluorosulfates have been synthesized *via* ^19^F/^18^F isotopic exchange at room temperature in only 30 s and **(B)** The same target can be made from the corresponding imidazylates at 100°C in 10 min. Neither have been tested in aqueous conditions.

In addition to arylsulfonylchlorides, arylfluorosulfates (**87**) have also been radiolabeled with fluorine-18. Remarkably, they can be rapidly radiolabeled through ^19^F/^18^F isotopic exchange using the traditional azeotropically dried [^18^F]KF/Kryptofix 222 in acetonitrile in as little as 30 s at room temperature (Figure 14) (Zheng et al., 2021). Furthermore, they show excellent stability *in vivo*. Recently, Kwon *et al.* also reported the synthesis of fluorine-18 radiolabeled aryl fluorosulfates from their aryl imidazylate precursors (**89**) (Kwon et al., 2020). In comparison, fluorination was performed at high temperatures but greater than 50% yields were frequently obtained with only a few exceptions (Figure 14).

## Conclusion

PET has emerged as a powerful imaging technique for the detection, diagnosis and staging of disease. With their high specificity and affinity for their molecular targets, peptide and protein biomarkers of disease have gained significant attention as potential imaging agents for targeted PET. In particular, there has been interest in the radiolabeling of these molecules with the radionuclide fluorine-18. For peptides which can withstand the harsh conditions required for C-F bond formation this can be achieve using conventional methods. Radiolabeling proteins with fluorine-18, on the other hand, is restricted by the low specific activities obtained from electrophilic fluorination and the low nucleophilicity of [^18^F]fluoride in aqueous media. As such, radiofluorination of proteins has primarily been accomplished through indirect labeling with prosthetic groups. These multi-step methods are not however, optimal for the short life of fluorine-18.

Recently, efforts have been centered around the exploration of inorganic approaches to radiolabel peptides and proteins with [^18^F]fluoride. As with traditional C-F bond formation these methods have been used in the indirect radiolabeling of proteins with fluorine-18. They have however, also inspired further research into the possibility of directly radiolabeling proteins with [^18^F]fluoride. This review has presented those examples, and their foundational reactions, based on the formation of fluoride bonds at boron, various metal complexes, silicon, phosphorus and sulfur. Challenges in rendering this chemistry generally compatible in water and on proteins remain, but this work provides an important basis for further study.

The most noteworthy of these challenges is to develop imaging agents which are both hydrolytic stable and allow for rapid and high yielding fluorination on proteins. As discussed in this review, steric effects play a large role in increasing the hydrolytic stability of fluoride bonds. Unfortunately, however, this can come at the expense of rapid fluorination and high yields. Furthermore, a number of examples rely on high temperatures or organic co-solvents with purely aqueous and ambient temperature fluorination reactions comparatively rare. If these methods of fluorination are to be used for the direct radiofluorination of proteins, mild conditions are required. As direct methods of protein labeling develop, the scope and precision of radiolabeled proteins may improve, which could create future opportunities in medical diagnosis.
